# P48 BLADDER score-guided diagnostic and antimicrobial stewardship in preoperative urine culturing

**DOI:** 10.1093/jacamr/dlag102.054

**Published:** 2026-06-26

**Authors:** S Harshitha, Afrin Siddique, Angel Jennifer, D Suresh Kumar

**Affiliations:** SRIHER, Chennai, Tamil Nadu, India; MMM Hospital, Chennai, Tamil Nadu, India; MMM Hospital, Chennai, Tamil Nadu, India; MMM Hospital, Chennai, Tamil Nadu, India; Best of IDs, Chennai, India

## Abstract

**Background:**

Routine preoperative urine culture screening in cardiac surgery is frequently performed without clinical indication, leading to the detection of asymptomatic bacteriuria and unnecessary antimicrobial use. Current practice lacks structured symptom-based risk assessment. The BLADDER score is a clinical tool designed to differentiate symptomatic urinary tract infection (UTI) from colonization; however, its role in improving diagnostic yield and reducing inappropriate testing and treatment in preoperative settings remains unclear. This study evaluated the burden of unnecessary urine cultures and antibiotic use and examined the potential role of BLADDER score–guided stewardship. The main objective of this study was to assess inappropriate urine culture utilization and antibiotic initiation, and to evaluate whether BLADDER score–based risk stratification improves diagnostic and antimicrobial stewardship.

**Methods:**

A retrospective observational study was conducted in a tertiary care cardiac hospital in South India, including 130 adult patients undergoing preoperative urine culture. Routine practice involved screening irrespective of symptoms. A weighted BLADDER score (dysuria=2 points; blood in urine, loss of control, abdominal pain, fever, and urinary frequency=1 point each) was retrospectively applied. Patients were stratified into high-risk (score ≥2) and low-risk (score <2) groups. Clinical indication, culture positivity, and antibiotic initiation were obtained from medical and microbiological records. Patients with incomplete data were excluded. Descriptive statistical analysis was used to compare diagnostic yield and estimate potentially avoidable testing and antibiotic use.

**Results:**

Of 130 urine cultures, 63.3% (n≈82) were performed without clinical indication. The overall positivity rate was 23.8% (n≈31). Routine screening accounted for 81.5% of cultures and demonstrated lower diagnostic yield. The BLADDER score identified a high-risk group with significantly higher positivity (41.5%) compared to 15.7% in low-risk patients. A notable proportion of positive cultures occurred in asymptomatic individuals, resulting in antibiotic initiation without clear clinical indication. Application of BLADDER score–guided criteria would have excluded most low-risk patients from testing, thereby reducing unnecessary cultures and avoidable antimicrobial use.

**Conclusions:**

A majority (63%) of preoperative urine cultures were performed without clinical indication, contributing to unnecessary antibiotic use. BLADDER score–guided stratification improves identification of clinically relevant infections and supports targeted testing. Despite its retrospective design, this study supports prospective implementation to strengthen diagnostic and antimicrobial stewardship.

**Table 1.** Comparison of routine practice and BLADDER score–guided approach

Parameter	Routine practice	BLADDER score approach
Total cultures	130	130 (reclassified)
Cultures without indication	63.3% (∼82)	Reduced (low-risk excluded)
Overall positivity	23.8% (∼31)	Higher yield in high-risk (41.5%)
Low-risk positivity	—	15.7%
Antibiotic use	Frequent in asymptomatic	Potentially reduced

